# Medicinal cannabis for tics in adolescents with Tourette syndrome

**DOI:** 10.1192/bjo.2025.35

**Published:** 2025-07-10

**Authors:** Valsamma Eapen, Ping-I Lin, Kaitlyn Taylor, Eunice Chan, Paul Chay, Noel Cranswick, Amy Ka, Feroza Khan, Jonathan M. Payne, Chidambaram Prakash, Ramya Velalagan, Daryl Efron

**Affiliations:** Discipline of Psychiatry, School of Clinical Medicine and Health, University of New South Wales, Sydney, NSW, Australia; Academic Unit of Child Psychiatry Southwest Sydney, South Western Sydney Local Health District and Ingham Institute, Sydney, NSW, Australia; Centre for Community Child Health, Murdoch Children’s Research Institute, Parkville, VIC, Australia; Department of General Medicine, Royal Children’s Hospital, Parkville, VIC, Australia; Department of Paediatrics, University of Melbourne, Melbourne, VIC, Australia

**Keywords:** Tic disorders, medicinal cannabis, paediatric neurology, tics, clinical trials

## Abstract

Medicinal cannabis has been trialled for Tourette syndrome in adults, but it has not been studied in adolescents. This open-label, single-arm trial study evaluated the feasibility, acceptability and signal of efficacy of medicinal cannabis in adolescents (12–18 years), using a Δ9-tetrahydrocannabinol:cannabidiol ratio of 10:15, with dose varying from 5 to 20 mg/day based on body weight and response. The study demonstrated feasibility of recruitment, acceptability of study procedures, potential benefits and a favourable safety profile, with no serious adverse events. Commonly reported adverse events were tiredness and drowsiness, followed by dry mouth. Statistically significant improvement was observed in parent and clinician reports on tics (paired *t*-test *P* = 0.003), and behavioural and emotional issues (paired *t*-test *P* = 0.048) and quality of life as reported by the parent and young person (paired *t*-test *P* = 0.027 and 0.032, respectively). A larger-scale, randomised controlled trial is needed to validate these findings.

Tourette syndrome significantly affects daily functioning and quality of life (QoL) for young people and their families.^1^ Although psychotropic medication and behavioural therapies can assist with symptom control,^2^ treatment response is variable^3^ and side-effects are common, which can affect medication adherence.^4^ Alternative interventions are critically needed, and medicinal cannabis has emerged as a potential option in a few studies in adults, with varying results;^5,6^ however, the efficacy and safety in young people is unknown.^7^

The main objective of this pilot study was to conduct the first evaluation of the feasibility of recruitment, protocol adherence, acceptability of study procedures and signal of efficacy of medicinal cannabis in a Δ9-tetrahydrocannabinol:cannabidiol ratio of 10:15 for tic symptoms and QoL in adolescents with Tourette syndrome. The secondary objective was to assess its safety and side-effects. The overall goal was to inform future randomised controlled trials.

## Method

This was an open-label, single-arm trial of medicinal cannabis, with participants with a body weight <50 kg receiving a maximum Δ9-tetrahydrocannabinol dose of 10 mg/day and those with a body weight ≥50 kg receiving a maximum Δ9-tetrahydrocannabinol dose of 20 mg/day.

This study was conducted in accordance with the Consolidated Standards of Reporting Trials guidelines, and ethical approval was granted from the South-Western Sydney Local Health District Ethics Committee (approval number 2021/ETH11096). It was registered with the Australian and New Zealand Clinical Trials Registry (identifier ACTRN12622000031763).

Adolescents aged 12–18 years, with a DSM-5 diagnosis of Tourette syndrome, a total tic severity score of ≥20 on the Yale Global Tic Severity Scale (YGTSS)^8^ and no change to medication/interventions for the previous 4 weeks were enrolled from 14 April 2022 to 19 October 2023. All participants had been treated with psychotropic medication (and six had also received behavioural treatments), but had failed to achieve meaningful improvement despite appropriate trial at therapeutic doses and duration. All parents and participants (aged >16 years) provided written informed consent. Exclusion criteria included non-English-speaking parents, a personal/family history of serious mental disorders such as psychosis, abnormal liver function, recent illicit drug or certain medication use, pregnancy or breastfeeding, and clinically significant suicidal ideation in the previous 12 months.

Medicinal cannabis oil with a concentration of Δ9-tetrahydrocannabinol 10 mg/mL and cannabidiol 15 mg/mL (Cann Group, Australia) was used.

The dose was stratified by participant weight (<50 kg and ≥50 kg) at enrolment. The titration schedule included an initial dose of 1 mg/day, followed by uptitration over 21 days by 1 mg every 4–5 days to reach 5 mg/day for participants weighing <50 kg, and 1 mg every 2–3 days to reach 10 mg/day for participants weighing ≥50 kg by day 29. This was administered once daily, with a recommendation for it to be taken in the evening, except when twice a day divided dosage was used where side-effects were experienced. Participants achieving a 55% reduction from baseline on the Parent Tic Questionnaire (PTQ) and a favourable Clinical Global Impression – Improvement score (1 or 2) with tolerable side-effects were considered responders and stayed on this dose until day 85. Non-responders continued uptitration over 21 days, to 10 mg (<50 kg) or 20 mg (≥50 kg).

Tics were evaluated using the clinician-administered YGTSS^8^ and parent-administered PTQ.^9^ Acceptability and adverse events were ascertained using a weekly log and the Modified Liverpool Adverse Events Profile (LAEP) questionnaire.^10^ Clinicians completed Clinical Global Impression – Severity and Improvement scales and the Premonitory Urge for Tics Scale (PUTS).^11^ Tics and associated features and comorbid conditions were assessed by a clinician, using the National Hospital Interview Schedule (NHIS),^12^ and parents completed the ADHD Rating Scale,^13^ the Strengths and Difficulties Questionnaire (SDQ)^14^ for behavioural and emotional issues, and the Gilles de la Tourette Syndrome-Quality of Life Scale (GTS-QOL).^15^ Urine testing for illicit drugs and blood tests for urea and electrolytes at screening and liver function during screening and on day 85 were done. See Supplementary Table 1 for the schedule of activities.

We conducted equivalence tests for tic-related symptoms and QoL by using paired *t*-tests with two-sided *P*-values. Comparisons of baseline features for responders and non-responders, as well as experience of adverse events, were conducted with Mann–Whiney *U*-tests. Effect sizes were estimated for both types of statistical tests. Given that this is a feasibility study, we employed exploratory analyses designed to guide the development of a larger trial. To align with the study’s preliminary nature, these analyses did not account for covariates, and adjustments for multiple testing were not performed.

## Results

The mean age of the ten participants was 14.4 years (range 12–18 years, s.d. = 1.71). As per NHIS score at baseline, attention-deficit hyperactivity disorder (ADHD) was present in six (60%) participants, obsessive–compulsive disorder was present in five (50%) participants, anxiety was present in nine (90%) participants, autism was present in four (40%) participants, intellectual disability was present in three (30%) participants and depression was present in three (30%) participants. The feasibility of recruitment was demonstrated, and the study procedures were found to be acceptable as evidenced by 100% attendance at clinic visits and completion of questionnaires on time, as per protocol. One participant (non-responder at day 29) withdrew on day 49, as the family went on overseas holidays and the medicinal cannabis could not be continued. Of the ten participants, seven did not respond to the low dose, necessitating uptitration. Although two had adverse events necessitating reduction back to the low dose, no participants experienced any serious adverse events. There was no difference between responders and non-responders in terms of needing uptitration to the higher dose. The most commonly reported adverse events were blurred vision, dry mouth, increased appetite and decreased motivation (22% for all). These were followed by unsteadiness, restlessness, headache, concentration, shaky hands, weight gain, dizziness, sleepiness, weight loss, confusion, euphoria and disorientation (11% for all), based on a two-point change in LAEP score at each symptom level. Side-effects reported outside of those determined by the LAEP were tiredness and drowsiness (40%), followed by dry mouth (30%). Of those who reported drowsiness, one reported this at days 22 and 29 (on the lower dose), but as it did not interfere with their activities, no dose change was made. Three patients reported drowsiness after uptitration of the dose (non-responders); two improved after splitting into twice daily dosage, whereas the other improved following reduction in dose.

There was positive signal of efficacy with a statistically significant improvement in parent and self-reported tics and QoL, as well as behavioural/emotional issues as per the SDQ (Table 1). The clinician-reported YGTSS impairment scores (Supplementary Fig. 1) and parent-reported severity scores remained statistically significant even under a conservative Bonferroni correction (adjusted threshold *P* < 0.005). Premonitory urges and ADHD symptoms showed no significant difference. The baseline SDQ score was associated with treatment response (*P* = 0.0236), and baseline GTS-QOL score also showed a marginal association with treatment response, indicating a potential role of baseline characteristics in outcomes. The adverse events LAEP score on day 85 did not differ between responders and non-responders (*z* = 1.03, *P* = 0.3051, *r* = 0.33). The effect size was estimated to be 1.37 for the YGTSS data, leading to a power of 0.84. See Supplementary Table 2 for details.

**Table 1:** Results of paired *t*-test analyses for the treatment efficacy corresponding to different indicators

Outcome	Baseline mean (s.d.)	Day 29 mean (s.d.)	Day 85 mean (s.d.)	Day 29 versus baseline	Day 85 versus baseline
Tic severity (clinician rated), YGTSS impairment	74.11 (5.68)		45.33 (8.38)		*P* = 0.0035, *d* = 1.36
Tic severity (parent reported), YGTSS severity	35.22 (2.97)		20.89 (13.80)		*P* = 0.0160, *d* = 1.01
Tic severity (parent reported), PTQ^a^	69.80 (5.06)	43.70 (10.68)	40.11 (10.10)	P = 0.0141	*P* = 0.0038, *d* = 1.34
Quality of life, GTS-QOL (parent reported)	74.50 (7.40)		50.40 (8.05)		*P* = 0.0272, *d* = 0.83
Quality of life, GTS-QOL (self-reported)	76.70 (6.18)		54.50 (7.68)		*P* = 0.0326, *d* = 0.8
Quality of life, life satisfaction (parent reported)	41.75 (4.77)		54.50 (9.66)		*P* = 0.1094, *d* = 0.65
Quality of life, life satisfaction (child reported)	52.63 (4.95)		58.34 (8.93)		*P* = 0.4913, *d* = 0.26
ADHD symptoms, ADHD rating scale	32.78 (4.22)		25.44 (4.75)		*P* = 0.0631, *d* = 0.72
Emotional and behavioural problems, SDQ-difficulty	29.22 (2.12)		25.89 (1.67)		*P* = 0.0485, *d* = 0.78
Premonitory urge symptoms, PUTS	25.33 (2.13)		24.00 (1.64)		*P* = 0.6238, *d* = 0.17
Outcome	Baseline mean (s.d.)	Day 29 mean (s.d.)	Day 85 mean (s.d.)	Day 29 versus baseline	Day 85 versus baseline
Tic severity (clinician rated), YGTSS impairment	74.11 (5.68)		45.33 (8.38)		*P* = 0.0035, *d* = 1.36
Tic severity (parent reported), YGTSS severity	35.22 (2.97)		20.89 (13.80)		*P* = 0.0160, *d* = 1.01
Tic severity (parent reported), PTQ^a^	69.80 (5.06)	43.70 (10.68)	40.11 (10.10)	*P* = 0.0141	*P* = 0.0038, *d* = 1.34
Quality of life, GTS-QOL (parent reported)	74.50 (7.40)		50.40 (8.05)		*P* = 0.0272, *d* = 0.83
Quality of life, GTS-QOL (self-reported)	76.70 (6.18)		54.50 (7.68)		*P* = 0.0326, *d* = 0.8
Quality of life, life satisfaction (parent reported)	41.75 (4.77)		54.50 (9.66)		*P* = 0.1094, *d* = 0.65
Quality of life, life satisfaction (child reported)	52.63 (4.95)		58.34 (8.93)		*P* = 0.4913, *d* = 0.26
ADHD symptoms, ADHD rating scale	32.78 (4.22)		25.44 (4.75)		*P* = 0.0631, *d* = 0.72
Emotional and behavioural problems, SDQ-difficulty	29.22 (2.12)		25.89 (1.67)		*P* = 0.0485, *d* = 0.78
Premonitory urge symptoms, PUTS	25.33 (2.13)		24.00 (1.64)		*P* = 0.6238, *d* = 0.17

Effect size expressed as the Cohen’s *d*. YGTSS, Yale Global Tic Severity Scale; PTQ, Parent Tic Questionnaire; GTS-QOL, Gilles de la Tourette Syndrome Quality of Life; ADHD, attention-deficit hyperactivity disorder; SDQ, Strengths and Difficulties Questionnaire.; PUTS, Premonitory Urge for Tics Scale.

a No difference between days 29 and 85 (*P* = 0.5354).

## Discussion

The results of this study, the first of its kind in adolescents with Tourette syndrome, has provided preliminary evidence of feasibility and acceptability of the study design for use of medicinal cannabis in adolescents, as well as indicating a potential favourable impact on tic symptoms and QoL. Although there is emerging evidence supporting the use of cannabis-based interventions in the management of Tourette syndrome in adults,^5,6^ this study uniquely contributes to the evidence on the benefits and safety of medicinal cannabis in adolescents with Tourette syndrome.

The identification of baseline SDQ difficulty score as a predictor for treatment response adds valuable insights, emphasising the importance of considering individual characteristics when tailoring treatment plans.

The absence of differences in the adverse events score between responders and non-responders raises interesting questions about the metabolism and pharmacological effects of medicinal cannabis, and its link to treatment outcomes. The long-term impact of medicinal cannabis on neurodevelopmental trajectories also deserves further exploration.

The findings of this study should be interpreted with caution because of the study limitations related to the small sample size and lack of controls, and the open-label uncontrolled nature of the study. However, having demonstrated the recruitment feasibility, acceptability of study procedures, potential benefits and favourable safety profile, this study paves the way for larger randomised controlled trials to validate the findings.

## Supporting information

Eapen et al. supplementary material 1 Eapen et al. supplementary material

Eapen et al. supplementary material 2 Eapen et al. supplementary material

Eapen et al. supplementary material 3 Eapen et al. supplementary material

## Data Availability

Data are available upon request. No patients were involved in the design or conduct or reporting or dissemination plans of our research.

## Supplementary material

The supplementary material can be found at https://doi.org/10.1192/bjo.2025.35
