# Supplementary figures and images for: Medicinal cannabis for tics in adolescents with Tourette syndrome
Source: BJPsych Open. 2025 Jul 10;11(4):e145. doi: 10.1192/bjo.2025.35 (PMC12247065; doi:10.1192/bjo.2025.35)

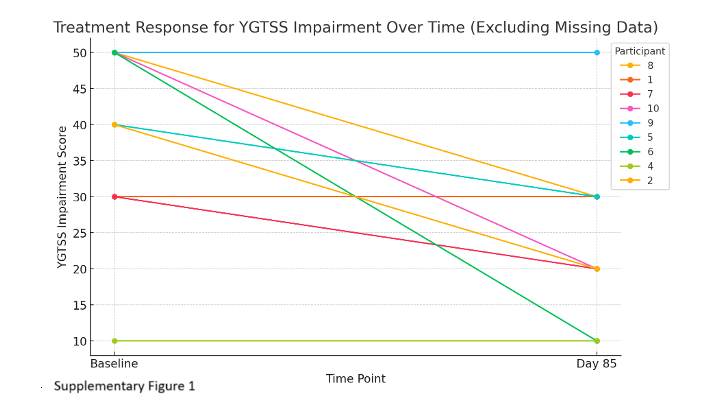

Supplement: Eapen et al. supplementary material 1 — Eapen et al. supplementary material [file S2056472425000353sup001.zip › Supplementary Figure 1 New 3.9.24.png]
