# Supplementary material for: Medicinal cannabis for tics in adolescents with Tourette syndrome
Source: BJPsych Open. 2025 Jul 10;11(4):e145. doi: 10.1192/bjo.2025.35 (PMC12247065; doi:10.1192/bjo.2025.35)
Supplement: Eapen et al. supplementary material 2 — Eapen et al. supplementary material [file S2056472425000353sup002.docx]

Supplementary Table 1: Schedule of activities and measures

|  | Screening | Treatment Phase | | | Last dose  D91 | 28 day follow up post drug cessation (D119)  +/-3 days |
| --- | --- | --- | --- | --- | --- | --- |
|  |  | D1 | D29  +/- 3 days | End of Treatment D85 ±4 |  |  |
| Clinic Visit (CV) or Phone call (PC) | CV | CV/PC | PC | CV | CV/PC | PC |
| Confirm eligibility | X |  |  |  |  |  |
| Yale Global Tic Severity Rating Scale (YGTSS) | X |  |  | X |  |  |
| Parent Tic Questionnaire | X |  | X | X |  |  |
| Clinical Global Impression – Severity and Improvement scales | X |  | X | X |  |  |
| Premonitory Urge for Tics Scale (PUTS) | X |  |  | X |  |  |
| National Hospital Interview Schedule for Tics and related behaviors (NHIS) | X |  |  | X |  |  |
| ADHD rating scale | X |  |  | X |  |  |
| Strengths & Difficulties Questionnaire | X |  |  | X |  |  |
| Gilles de la Tourette Syndrome-Quality of Life Scale | X |  |  | X |  |  |
| Medical history | X |  |  |  |  |  |
| Physical examination | X |  |  | X |  |  |
| Heart Rate, Blood Pressure and Weight | X |  |  | X |  |  |
| Concomitant medications | X | X |  | X |  | X |
| Blood test (urea and electrolytes, FBC) | X |  |  |  |  |  |
| Blood test (Liver function tests) | X |  |  | X |  |  |
| Urine test* | X |  |  | X |  |  |
| Dispense study medication |  | X |  |  |  |  |
| Study drug administration |  | X------------------------------X | | | |  |
| Dispense diary cards |  | X |  |  |  |  |
| Collect diary cards |  |  |  | X | X |  |
| Dose assessment |  |  | X |  |  |  |
| Liverpool Adverse Event Profile (LAEP) | X |  |  | X |  |  |
| Compliance check |  |  |  | X | X |  |
| Safety check phone call |  |  | X |  |  | X |
| Pilot evaluation questionnaire |  |  |  |  |  | X |

** Illicit drug test for all participants, and pregnancy test for women of childbearing potential only*
