# Supplementary material for: Medicinal cannabis for tics in adolescents with Tourette syndrome
Source: BJPsych Open. 2025 Jul 10;11(4):e145. doi: 10.1192/bjo.2025.35 (PMC12247065; doi:10.1192/bjo.2025.35)
Supplement: Eapen et al. supplementary material 3 — Eapen et al. supplementary material [file S2056472425000353sup003.docx]

Supplementary table: Total number of participants who completed LEAP (Parent) at screening and Day 85 visits: 9

Number of participants with 2-point change at each symptom level:

|  |  |  |  |  |  |  |  |  |  |
| --- | --- | --- | --- | --- | --- | --- | --- | --- | --- |

| **Symptoms** | **Number of children** | **Percentage of children reporting on LAEP** |
| --- | --- | --- |
| Unsteadiness | 1 | 11 |
| Restlessness | 1 | 11 |
| Headache | 1 | 11 |
| Blurred vision | 2 | 22 |
| Concentration problem | 1 | 11 |
| Dry mouth | 2 | 22 |
| Shaky hands | 1 | 11 |
| Weight gain | 1 | 11 |
| Dizziness | 1 | 11 |
| Sleepiness | 1 | 11 |
| Gain of appetite | 2 | 22 |
| Weight loss | 1 | 11 |
| Motivation problem | 2 | 22 |
| Confusion | 1 | 1 |
| Euphoria | 1 | 1 |
| Disorientation | 1 | 1 |
